# Omental Torsion After Repeated Abdominal Blunt Trauma

**DOI:** 10.5812/atr.6881

**Published:** 2012-08-21

**Authors:** Mehrdad Hosseinpour, Azadeh Abdollahi, Hoda Jazayeri, Hamid Reza Talari, Ahmad Sadeghpour

**Affiliations:** 1 Trauma Research Center, Kashan University of Medical Sciences, Kashan, IR Iran

**Keywords:** Acute Abdomen, Greater Omentum, Trauma, Omental Torsion

## Abstract

Omental torsion is caused by the rotation of the greater omentum on its axis which may lead to tissue infarction and necrosis. It is a rare cause of acute abdomen. Signs, symptoms and paraclinical data are not specific. The patients usually undergo laparotomy for acute abdomen of poorly defined origin. High index of suspicious is required for the diagnosis of this entity. The diagnosis is usually confirmed after an explorative laparotomy. We present clinical characteristics and imaging findings of omental torsion in a young man following repeated blunt abdominal trauma.

## 1. Introduction

Omental torsion is a type of volvulus created by the rotation of the omentum on its long axis, which can lead to tissue ischemia and necrosis. Torsion of the omentum is a rare pathology in which the omentum twists until its vascularity is compromised (1, 2). The clinical presentation mimics the common causes of acute surgical abdomen. It occurs in men (2) and in third and fourth decades of life but can occur at any age (3), so that 0.05 to 0.1% were diagnosed in children during surgery and mostly affected children between9 and 16 years old (4, 5). Torsion of the omentum can be primary or secondary. Secondary torsion is associated with hernias, tumors and adhesions (6). Primary omental torsion is described when there are no known causes but several anatomic defects of the omentum such as bifid omentum and accessory omental tissue have been mentioned (6). Some predisposing factors are obesity, gender, sudden strong increase in intra-abdominal pressure and trauma.

## 2. Case report

**Figure 1.**
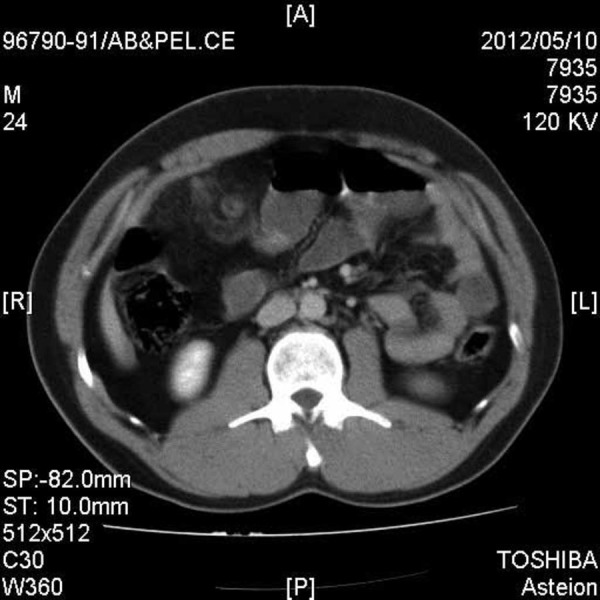
Abdominal CT Scan

A thirty year old man was admitted to the Emergency Department of Shahid Beheshti university hospital with the chief complaint of abdominal pain, which occurred after jumping his one year old daughter on his abdomen. The patient also noted another blunt abdominal trauma, due to a car turn-over, 20 days previously. The pain was sustained and felt in the peri-umbilical region. In the physical examination; blood pressure was 120/70 mmHg, pulse rate was 86, respiratory rate was 12 and the patient’s oral temperature was 36.8° C. The abdomen was diffusely tender in deep palpation without distention, rigidity or guarding. In the laboratory data; white blood cell (WBC) count of 9000, neutrophil count at 65%, Hemoglobin (HB) was 13.3 g/dL and Urine analysis was normal. Ultrasonography showed increased echogenicity of mesentery without free fluid in the abdomen cavity. An abdominal Computerized tomography (CT) scan revealed increased attenuation in the small bowel mesenteric fat. No vein or artery was detected in the mesenteric (Figure 1).

**Figure 2.**
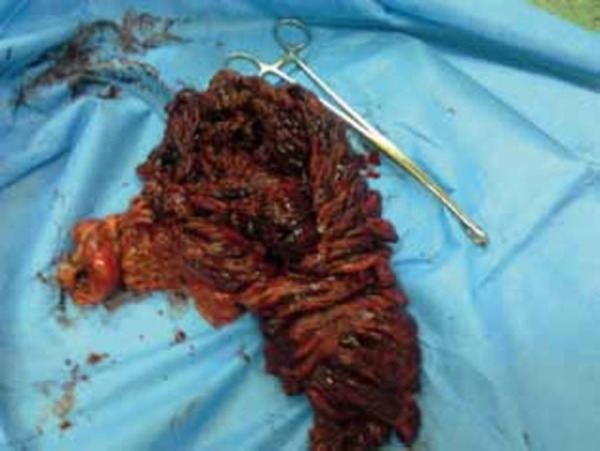
Greater Omentum Twisted Around its Basal Axis

The patient was observed with conservative management. After 12 hours, we noted an increase in abdominal tenderness, with rebound tenderness and guarding. Blood Pressure = 130/75 mmHg, Pulse Rate = 110 beat/min, Oral temperature = 38 ºC, WBC = 20.340 X 10^3^/μL, neutrophils = 83%, and HB = 12.5 g/dL. Therefore, we decided to perform an explorative laparotomy, and a midline laparotomy was subsequently performed. There was hemorrhagic fluid collecting in the peritoneal cavity. The greater omentum was twisted around its basal axis and it was black, showing necrosis (Figure 2). Other abdominal organs were normal. An omentectomy was performed and peritoneal lavage was also performed. Histopathological study revealed an infarcted omentum. On the third postoperative day, oral feeding was started. The patient tolerated the food well and he was discharged in a good general condition on the fifth day after the operation.

## 3. Discussion

The first case of omental torsion was described in 1896, and fewer than 300 cases have been reported. The majority of patients (85%) were adults and the remaining 15% were found in the pediatric population (2). Most patients are male in the third and fourth decades of life (4). Omental torsion can be primary or secondary due to; acute cholecystitis, pancreatitis, adnexitis, tumors, appendix epiploica, diverticulitis, or post-surgical adhesions (1). Other etiologies are; obesity, strong sudden increase in intra-abdominal pressure brought on by coughing or violent exercise, trauma, larger than normal or twisted epiploic blood vessels, accelerated peristalsis, or some acute process in an intracavitary organ that causes the migration of a segment of the omentum to the affected site. Torsion of the greater omentum is a rare cause of acute abdomen and it is often misdiagnosed as; acute appendicitis, acute cholecystitis, epiploic appendagitis, and various other diseases (7). The greater omentum is a four-layered fatty sheet of peritoneum that suspends from the greater gastric curvature, surrounding and protecting internal organs, with attachments to the diaphragm (8). Although omental torsion can involve both sides of the abdomen, a right-sided omental torsion is much more common than a left-sided one, because the right part of the omentum is longer and more mobile than the left side (9-11). Torsion occurrence on the right side may mimic symptoms of; perforated duodenal ulcer, acute appendicitis, acute cholecystitis, cecal diverticulitis or epiploic appendagitis (12). The primary symptom is pain, which is frequently localized in the right lower quadrant of the abdomen. The onset of pain is usually sudden and it does not radiate to the abdominal wall (9, 13, 14). Abdominal examination reveals right-sided tenderness, guarding and rebound tenderness (15, 16). A diagnosis based on clinical signs and physical examination alone is difficult and in most of the reported cases it was established with a laparotomy. Nausea and vomiting occur in less than 50% of cases, leuckocytosis and fever is common. Abdominal tenderness and peritonism is always present. If a large segment of the omentum is involved, a mass may be felt (17). In general, patients with omental torsion compared to patients with acute appendicitis are less unwell, and the duration of the disease extends over a longer period of time (18). Compared to appendicitis, torsion has an incidence of 0.0016 – 0.37%, which is a ratio of less than 4 cases per 1 000 cases of appendicitis (19). Due to the similarity of symptoms with other conditions, ultrasound (US) and computerized tomography (CT) scans are often performed to assist in the diagnosis. US findings in omental torsion usually include hyperechoic, as was observed in our patient. Other findings include a noncompressible, ovoid intra-abdominal mass adherent to the abdominal wall, which is located in the umbilical region or anterolaterally to the right half of the colon. US findings can rule out acute cholecystitis. By showing a normal appearing gallbladder, appendix and no signs of diverticulitis, the CT scan will help in the differential diagnosis (12, 20). The larger size and medial location of the mass relative to the ascending or descending colon favors omental abnormality, rather than primary epiploic appendagitis, in which the omental abnormality is typically related to the colon. Pictures of a fatty like mass can be seen in; lipoma, liposarcoma, angiomyolipoma, teratoma, mesenteric lipodystrophy, pseudomyxoma peritonei and segmental infarction of the omentum (12, 20), however, specific CT findings in omental torsion include diffuse streaking in a whirling pattern of fibrous and fatty folds, namely concentric linear strands that are not present in other omental diseases, however, it is not specific enough to make a diagnosis and show linear, nodular and branching soft tissue structures (1, 21, 22). Due to the difficulty in finding clear imaging and the similarity of the clinical presentation with other diseases along with the low incidence rates, a preoperative diagnosis is rare, involving only 0.6 – 4.8% of cases (20). Abe et al. presented a case report of torsion of the greater omentum, which was diagnosed by CT multi-planar reconstruction (MPR) preoperatively and it seems that MPR can be a useful imaging tool in making a diagnosis (7). Treatment consists of resecting the infracted omentum. In secondary omental torsion, the underlying etiologic condition must be treated as well (1). Laparoscopic surgery is an alternative treatment of choice. Successful conservative treatment has been reported in only seven cases of segmental omental infarction, and these eventually atrophied and formed fibrosis on radiological follow-up (19).

Omental torsion is not a common pathology, but it should be considered in the differential diagnosis of acute abdomen, particularly in patients with a history of repeated blunt abdominal trauma.
